# microRNA-203 suppresses invasion and epithelial-mesenchymal transition induction via targeting NUAK1 in head and neck cancer

**DOI:** 10.18632/oncotarget.6972

**Published:** 2016-01-22

**Authors:** Mariko Obayashi, Maki Yoshida, Takaaki Tsunematsu, Ikuko Ogawa, Tomonori Sasahira, Hiroki Kuniyasu, Issei Imoto, Yoshimitsu Abiko, Dan Xu, Saori Fukunaga, Hidetoshi Tahara, Yasusei Kudo, Toshitaka Nagao, Takashi Takata

**Affiliations:** ^1^ Department of Oral and Maxillofacial Pathobiology, Institute of Biomedical & Health Sciences, Hiroshima University, Hiroshima, Japan; ^2^ Department of Oral Molecular Pathology, Institute of Health Biosciences, The University of Tokushima Graduate School, Tokushima, Japan; ^3^ Center of Oral Clinical Examination, Hiroshima University Hospital, Hiroshima, Japan; ^4^ Department of Molecular Pathology, Nara Medical University School of Medicine, Nara, Japan; ^5^ Department of Human Genetics, Institute of Health Biosciences, The University of Tokushima Graduate School, Tokushima, Japan; ^6^ Department of Biochemistry, School of Dentistry at Matsudo, Nihon University, Chiba, Japan; ^7^ Department of Cellular and Molecular Biology, Graduate School of Biomedical Sciences, Hiroshima University, Hiroshima, Japan; ^8^ Institute of Environmental Systems Biology, Dalian Maritime University, Dalian, China; ^9^ Department of Anatomic Pathology, Tokyo Medical University, Tokyo, Japan

**Keywords:** invasion, microRNA (miRNA), epithelial-mesenchymal transition (EMT), head and neck squamous cell carcinoma (HNSCC), microarray

## Abstract

Head and neck squamous cell carcinoma (HNSCC) has a high capacity for invasion. To identify microRNAs (miRNAs) that regulate HNSCC invasion, we compared miRNA expression profiles between a parent HNSCC cell line and a highly invasive clone. The miR-200 family and miR-203 were downregulated in the clone. Here we focused on the role of miR-203 in invasion and epithelial-mesenchymal transition (EMT) induction in HNSCC. miR-203 was downregulated during EMT induction. Moreover, ectopic overexpression of miR-203 suppressed the invasion and induced mesenchymal-epithelial transition (MET) in HNSCC cells. Interestingly, we identified NUAK family SNF1-like kinase 1 (NUAK1) as a novel target gene of miR-203 by cyclopedic analysis using anti-Ago2 antibody. Increased expression of NUAK1 was observed during EMT induction, and ectopic expression of miR-203 delayed EMT induction by suppressing NUAK1 expression. Moreover, NUAK1 overexpression promoted the invasion of HNSCC cells. Importantly, NUAK1 expression was well correlated with poor differentiation, invasiveness, and lymph node metastasis in HNSCC cases. Overall, miR-203 has a tumor-suppressing role in invasion and EMT induction by targeting NUAK1 in HNSCC, suggesting miR-203 as a potential new diagnostic and therapeutic target for the treatment of HNSCC.

## INTRODUCTION

Head and neck squamous cell carcinoma (HNSCC) is one of the most common types of human cancer, with an annual incidence of more than 500,000 cases worldwide [1]. HNSCC is associated with severe disease- and treatment-related morbidity, and has a 5-year survival rate of approximately 50%; this rate has not improved in more than two decades [2]. Cancerous metastasis is the most important prognostic factor of HNSCC as in other carcinomas [3]. Like most epithelial cancers, HNSCC results from the accumulation of genetic and epigenetic alterations in a multistep process. In early metastasis, cancer cells experience reduced cell-to-cell adhesion and increased mobility through epithelial-mesenchymal transition (EMT), by which the phenotype of the cells changes from epithelial to mesenchymal [4]. During EMT, cancer cells become detached from each other. This results from decreased E-cadherin expression due to hypermethylation of the gene's promoter region, or transcriptional repression caused by zinc finger E-box-binding homeobox 1 and 2 (ZEB1, ZEB2), Snail family zinc finger 1 and 2 (SNAI1, SNAI2), and Twist family bHLH transcription factor 1 (TWIST) [5,6].

microRNAs (miRNAs) are a class of highly conserved, 18-25-nucleotide, small non-coding RNAs that decrease the expression of certain genes through translational repression or mRNA degradation. They play important roles not only in various biological processes including cell proliferation, stress resistance, and metabolism, but also in pathogenesis. Recently, many reports have shown that several miRNAs have oncogenic or tumor-suppressive activities [7,8]. Of these, members of the miR-200 family (miR-200a, -200b, -200c, -141, and -429) have shown the greatest activity during EMT [9]. The miR-200 family directly suppresses ZEB1/ZEB2 and maintains E-cadherin expression [10–12].

In a previous study, we established MSCC-1 cells from a cervical lymph node metastasis of gingival squamous cell carcinoma (SCC) [13]. Subsequently, a highly invasive MSCC-inv1 clone was isolated from MSCC-1 cells using an *in vitro* invasion assay [14]. Moreover, we identified several molecules including periostin by comparing the transcriptional profiles of MSCC-1 and MSCC-inv1 [15]. Interestingly, MSCC-inv1 has EMT features such as spindle shape and decreased E-cadherin expression compared with parental MSCC-1. Here, we compared the miRNA expression profiles between these two cell lines to identify the microRNAs that differ in their expression. We identified the miR-200 family and miR-203 as having the most downregulated expression in the highly invasive clone. Because it is well known that the miR-200 family plays an important role in invasion and EMT in cancer, we focused on the role of miR-203 in EMT induction and invasion in HNSCC.

## RESULTS

### miR-203 and the miR-200 family are identified as downregulated genes in a highly invasive HNSCC cell line

**Figure 1:**
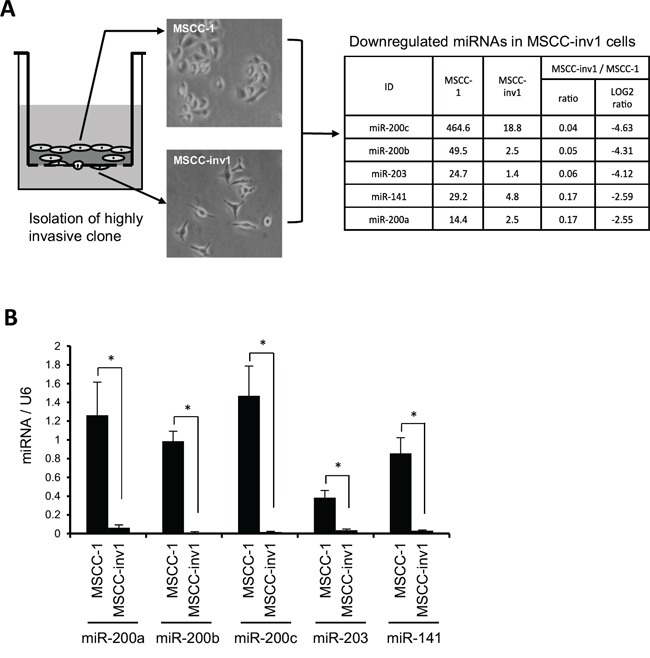
Identification of miR-200 family and miR-203 as candidate genes for suppression of invasion and/or EMT in HNSCC **A.** Schematic representation of miRNA expression profiles between parent cells (MSCC-1) and a highly invasive clones (MSCC-inv1). MSCC-inv1 cells were isolated from MSCC-1 cells by *in vitro* invasion assay. MSCC-inv1 cells are spindle shaped, while MSCC-1 cells are cobblestone-like shaped. The miRNA expression profile was examined by microarray. The table shows the top five downregulated miRNAs in MSCC-inv1 cells in comparison with MSCC-1 cells. **B.** Expression of the top five downregulated miRNAs in MSCC-inv1 cells was confirmed by real-time PCR. The graph shows the expression of these miRNAs (miRNA/U6) in MSCC-1 and MSCC-inv1 cells. All results are presented as means ± SD. *P < 0.05.

**Figure 2:**
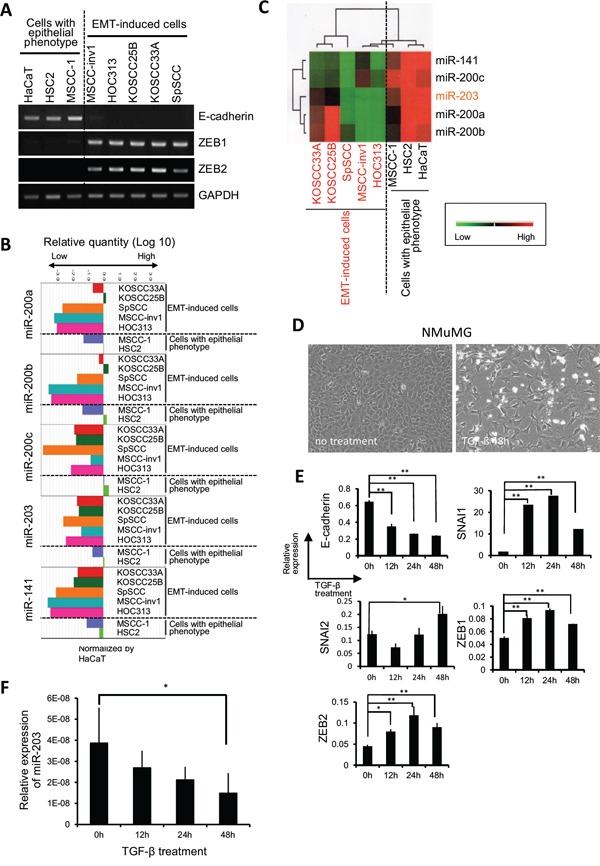
miR-200 family and miR-203 expression are correlated with EMT-induced phenotype in HNSCC **A.** Expression of E-cadherin, ZEB1, and ZEB2 was examined by RT-PCR in cells with epithelial phenotype (HaCaT, HSC2, and MSCC-1) and in EMT-induced cells (MSCC-inv1, HOC313, KOSCC25B, KOSCC33A, and SpSCC). GAPDH was used as a control. **B.** Expression of miR-200a, -200b, -200c, -141, and -203 was examined by real-time PCR in cells with epithelial phenotype (HaCaT, HSC2, and MSCC-1) and in EMT-induced cells (MSCC-inv1, HOC313, KOSCC25B, KOSCC33A, and SpSCC). Expression of these miRNAs in HNSCC cells was normalized by that in normal keratinocytes (HaCaT). The graph shows the relative quantity of miRNAs (miR-200a, -200b, -200c, -203, and -141). **C.** The heat map of the miRNA expression of the experiment described above is shown. **D.** NMuMG cells were treated with 10 ng/mL of TGF-β. The figure shows the cell shape at 0 (no treatment) and 48 h after TGF-β treatment. **E.** Expression of E-cadherin, SNAI1, SNAI2, ZEB1, and ZEB2 mRNA was examined by real-time PCR at 0, 12, 24, and 48 h (n = 3) after treatment with 10 ng/mL of TGF-β in NMuMG cells. The graph shows the expression of these mRNAs (mRNA/GAPDH). All results are presented as means ± SD. **P < 0.01, *P < 0.05. **F.** Expression of miR-203 was examined by real-time PCR at 0, 12, 24, and 48 h (n = 3) after treatment with 10 ng/mL of TGF-β in NMuMG cells. The graph shows the expression levels of miR-203 normalized by U6. The results are presented as means ± SD and *P < 0.05.

We compared the miRNA expression profiles between a parent cell line (MSCC-1) and a highly invasive clone (MSCC-inv1) by microarray analysis to identify genes that differed in their expression (Figure 1A). Several miRNAs were selectively downregulated in the clone (Figure 1A and Supplementary data 1). Among these genes, the miR-200 family (miR-200a, -200b, -200c, and -141) and miR-203 were included. We then confirmed the expression of these miRNAs in MSCC-1 and MSCC-inv1 cells (Figure 1B). We examined the expression of the miR-200 family (miR-200a, -200b, -200c and -141) and miR-203 in cells with the epithelial phenotype (HaCaT, HSC2, and MSCC-1) and EMT-induced cells (MSCC-inv1, HOC313, KOSCC25B, KOSCC33A, and SpSCC) by real-time PCR. EMT-induced cells, but not cells with the epithelial phenotype, showed no expression of E-cadherin and high expression of ZEB1 and ZEB2 (Figure 2A). In EMT-induced cells, all miRNAs tended to show lower expression levels in comparison with cells with the epithelial phenotype (Figure 2B). In particular, miR-200c, -203, and -141 were downregulated in all EMT-induced cells. Constructing a heat map from the results of real-time PCR, we identified similar expression tendencies between miR-141 and miR-200c, and between miR-200a and miR-200b (Figure 2C). It is worth noting that two pairs of miRNAs form clusters because their chromosomal sites are close and their seed sequences are similar. However, miR-203 showed a unique expression profile among these miRNAs.

We next examined expression of the miR-200 family (miR-200a, -200b, -200c, and -141) and miR-203 in an EMT-induction model using NMuMG cells. It has been reported that the miR-200 family is downregulated during EMT-induction [16]. After TGF-β treatment, NMuMG cells became spindle-shaped with decreased expression of E-cadherin and increased expression of E-cadherin repressors including SNAI1, SNAI2, ZEB1, and ZEB2 (Figure 2D and 2E). During EMT induction by TGF-β treatment, miR-203, miR-200a, -200b and -200c experienced time-dependent downregulation, but we did not detect miR-141 downregulation (Figure 2F and Supplemental Figure 1A). We transfected miR-200a, -200b, -200c, -141, and -203 into MSCC-inv1 cells. Transfection with all mature miRNAs induced mesenchymal-epithelial transition (MET) shown by increased expression of E-cadherin (Supplemental Figure 1B). Moreover, we removed each miRNA from transfection. Interestingly, all conditions of mature miRNAs transfection using four of five miRNAs induced MET, indicating that miR-200a, -200b, -200c, -141, and -203 play an important role in EMT induction in cooperation with each other (Supplemental Figure 1B). Because it is well known that the miR-200 family is involved in EMT [10–12], we focused on miR-203 as a candidate for an invasion- and/or EMT-suppressive miRNA in HNSCC cells.

### miR-203 suppresses the invasion of HNSCC cells *in vitro*

**Figure 3:**
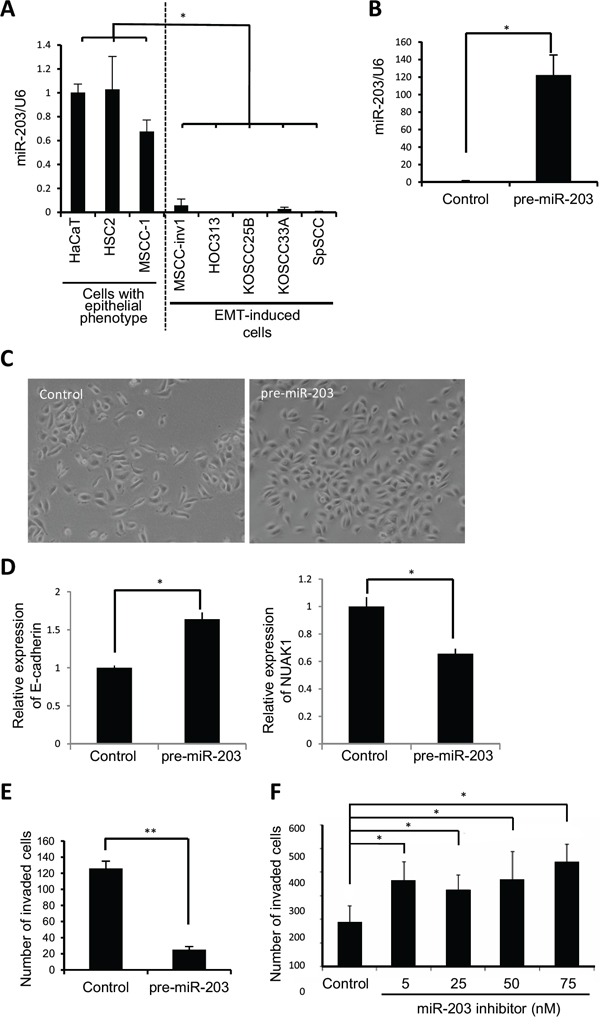
miR-203 suppresses cancerous invasion of HNSCC cells **A.** Expression of miR-203 was examined by real-time PCR in cells with epithelial phenotype (HaCaT, HSC2, and MSCC-1) and EMT-induced cells (MSCC-inv1, HOC313, KOSCC25B, KOSCC33A, and SpSCC). The graph shows miR-203/U6. The results are presented as means ± SD. *P < 0.05. **B.** Expression vector of pre-miR-203 or scramble negative control vector was transfected into MSCC-inv1 cells. miR-203/U6 was examined by real-time PCR and data were normalized to negative control-transfected samples. The results are presented as means ± SD. *P < 0.05. **C.** The figure shows the cell shape of control- and pre-miR-203-infected MSCC-inv1 cells. **D.** Expression of E-cadherin and NUAK1 mRNA was examined by real-time PCR in control- and pre-miR-203-infected MSCC-inv1 cells. The graph shows the expression of these mRNAs (mRNA/GAPDH). All results are presented as means ± SD. *P < 0.05. **E.** The graph shows invasion capability of negative control- or pre-miR-203-infected MSCC-inv1 cells. The invasiveness of the cells was determined by *in vitro* invasion assay for 9 h. **P < 0.01. **F.** HSC2 cells with miR-203 expression were transfected with miR-203 inhibitor (5, 25, 50, and 75 nM). The invasiveness of the cells was determined by *in vitro* invasion assay for 22 h. *P < 0.05.

We examined miR-203 expression in HNSCC cell lines. As shown in Figure 3A, miR-203 expression was significantly downregulated in EMT-induced HNSCC cells. To determine the role of miR-203 in HNSCC invasion, we transfected mature miR-203 into MSCC-inv1 cells. Although previous reports have shown that miR-203 suppresses cell proliferation in lung and esophageal cancer [17,18], miR-203 did not influence proliferation in MSCC-inv1 cells (Supplemental Figure 2A). Interestingly, miR-203 suppressed the invasion of MSCC-inv1 cells (Supplemental Figure 2B). To ensure that mature miRNA does not function at a supraphysiological level, we repeated these experiments using a miRNA vector that mimics miRNA biological processing. A pre-miR-203 lentiviral construct that stably expressed the miR-203 precursor in its native context was used to study the effect of miR-203 on MSCC-inv1 cell invasion (Figure 3B). Interestingly, pre-miR-203-infected MSCC-inv1 cells displayed a polygonal shape with upregulation of E-cadherin expression (Figure 3C and 3D). Pre-miR-203-infected MSCC-inv1 cells had lower invasion capability (Figure 3E). Moreover, to decrease endogenous miR-203 levels, HSC2 cells with epithelial phenotype were transfected with a specific miR-203 inhibitor. As shown in Figure 3F, the miR-203 inhibitor enhanced the invasion capability of the HSC2 cells. Collectively, these results suggest that miR-203 plays an important role in the invasion of HNSCC cells.

### NUAK1 is a target gene of miR-203

**Figure 4:**
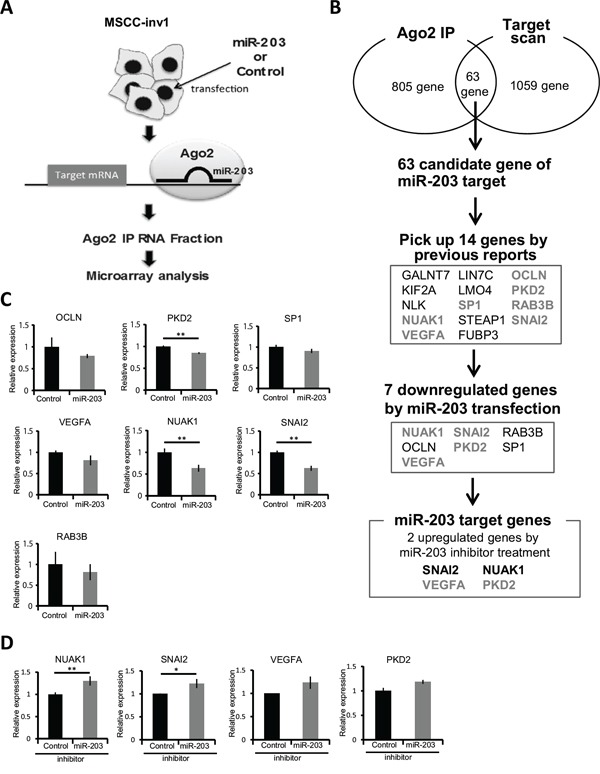
NUAK1 was identified as a target gene of miR-203 **A.** Schematic representation of the process of finding the target gene of miR-203. Mature miR-203 or control miRNA was transfected into MSCC1-inv1 cells. Then, miRNAs and target mRNAs were immunoprecipitated with anti-Ago2 antibody. Isolated mRNAs were analyzed by microarray. **B.** In MSCC-inv1 cells with ectopic miR-203 expression, 805 genes were enriched at least 2-fold over controls. By using Target Scan algorithm analysis (http://www.targetscan.org/), 1059 genes were predicted as target genes of miR-203. Sixty-three genes overlapped in the two analyses. Among those 63 genes, 14 were picked up by previous reports on the involvement of cancer, invasion, and EMT (Supplemental Table 1) and the result of Ago2-IP microarray. We then screened these genes by mature miR-203 transfection and miR-203 inhibitor transfection. Finally, we identified NUAK1 and SNAI2 as target genes of miR-203. **C.** The graph shows the expression of candidate genes of miR-203 (OCLN, PKD2, SP1, RAB3B, VEGFA, NUAK1, and SNAI2) in control- or mature miR-203-transfected MSCC-inv1 cells. The graph shows the expression of these mRNAs (mRNA/GAPDH). The results are presented as means ± SD. **P < 0.01. **D.** The graph shows the expression of NUAK1, SNAI2, VEGFA, and PKD2 in control- or miR-203 inhibitor-transfected HSC2 cells. The graph shows the expression of these mRNAs (mRNA/GAPDH). The results are presented as means ± SD. **P < 0.01, *P < 0.05.

Protein-coding mRNA transcripts that serve as target genes for miRNAs are bound indirectly to the Argonaute (Ago)-containing RNA-induced silencing complex (RISC). Therefore, to determine the target genes of miR-203, we used an antibody against wild-type human Ago2 to immunoprecipitate the RISC from the total cell lysate of MSCC-inv1 cells with or without ectopic miR-203 overexpression (Figure 4A). Ago2 is a member of the Ago family found in RISC complexes [19]. Because the available evidence suggests that binding among Ago family members is not sequence-specific [20,21], Ago2 binding is expected to serve as a proxy for binding with all Ago proteins. We prepared RNAs from immunoprecipitated extracts of control- or miR-203-transfected MSCC-inv1 cells with anti-Ago2 antibody for microarray analysis. In MSCC-inv1 cells with ectopic miR-203 expression, 805 genes were enriched at least 2-fold over the control cells (Figure 4B and Supplementary data 2). In addition, we searched the website, Target Scan Human 6.2 (http://www.targetscan.org/) for direct targets of miR-203 [22]. By using Target Scan algorithm analysis, 1059 genes are predicted as target genes of miR-203. Sixty-three genes overlapped between our analysis and the Target Scan algorithm analysis (Figure 4B). Among those 63 genes, 13 (GALNT7, KIF2A, NLK, NUAK1, VEGFA, LIN7C, LMO4, SP1, STEAP1, OCLN, PKD2, RAB3B, and SNAI2) are previously reported as a gene involved in cancer, invasion, and EMT, and FUBP3 showed considerable upregulation with miR-203 transfection in Ago2-IP analysis (Supplemental Table 1). Therefore, we focused on those 14 genes as candidates for miR-203 target genes (Figure 4B). SNAI1, SNAI2, ZEB2, and VEGFA have been reported as miR-203 targets related to cancer invasion [23–28]. Interestingly, SNAI2 and VEGFA were included in the 14 candidate target genes of miR-203. Among the 14 genes, 7 (NUAK1, VEGFA, SP1, OCLN, PKD2, RAB3B, and SNAI2) were downregulated by mature miR-203 transfection (Figure 4B, 4C, and Supplemental Figure 3A). Of those 7 genes, NUAK1, SNAI2, VEGFA, and PKD2 were upregulated by miR-203 inhibitor transfection (Figure 4B, 4D, and Supplemental Figure 3B). NUAK1 and SNAI2 were significantly upregulated (Figure 4D). NUAK1 expression in the MSCC-inv1 cells was slightly higher than in the MSCC-1 cells (Supplemental Figure 3C). SNAI2 expression in the MSCC-inv1 cells was similar to that in the MSCC-1 cells (Supplemental Figure 3C). SNAI2 has been identified as a target gene of miR-203 in breast and prostate cancer [26–28], and we confirmed that mature miR-203 decreased the relative luciferase activity of the 3′-UTR reporter vectors by approximately 50-70% (Supplemental Figure 4A). Pre-miR-203-infected MSCC-inv1 cells showed slightly lower expression of SNAI2 (Supplemental Figure 4B). Moreover, mature miR-203 transfection decreased the expression of SNAI2 in EMT-induced HNSCC cells (HOC313 and SpSCC) (Supplemental Figure 4C), and miR-203 inhibitor transfection increased the expression of SNAI2 in HSC2 cells of epithelial phenotype (Supplemental Figure 4D). Here, we focused on NUAK1 as a candidate novel target gene of miR-203.

**Figure 5:**
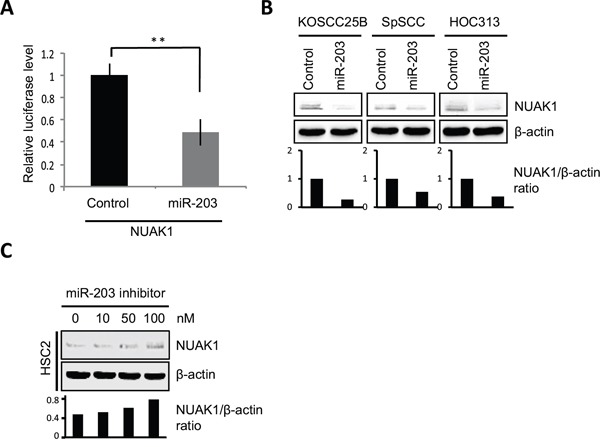
miR-203 suppresses NUAK1 expression **A.** Luciferase assays were performed with pmirGLO vector containing the 3′-UTR of NUAK1 and the graph shows the relative luciferase activity in mature miR-203- or control miRNA-transfected cells. The data were normalized to control samples and results are presented as means ± SD. **P < 0.01. **B.** NUAK1 expression was examined in control- or mature miR-203-transfected HNSCC cells (KOSCC25B, SpSCC, and HOC313) by western blotting analysis. The graph shows the NUAK1/β-actin ratio by densitometric analysis. **C.** NUAK1 expression was examined in control- or miR-203 inhibitor-transfected HSC2 cells by western blotting analysis. The graph shows the NUAK1/β-actin ratio by densitometric analysis.

To investigate whether miR-203 is able to directly target NUAK1 by interacting with its 3′-UTR *in vitro*, the 3′-UTR of NUAK1 was cloned and inserted downstream of a luciferase reporter gene. Subsequently, the mature miR-203 or control miRNA were cotransfected with 3′-UTR reporter vectors into 293T cells. The mature miR-203 decreased the relative luciferase activity of the 3′-UTR reporter vector by approximately 50% (Figure 5A). Indeed, mature miR-203 transfection decreased the expression of NUAK1 in EMT-induced HNSCC cells (KOSCC25B, SpSCC, and HOC313) (Figure 5B), and NUAK1 was downregulated in pre-miR-203-infected MSCC-inv1 cells (Figure 3D). Moreover, miR-203 inhibitor transfection increased the expression of NUAK1 in HSC2 cells of epithelial phenotype (Figure 5C). In the HNSCC cell lines, miR-203 expression was not always inversely correlated with NUAK1 expression, but inverse correlation between miR-203 and NUAK1 was observed in MSCC-1, HOC313, KOSCC25B, and SpSCC (data not shown). Thus, we demonstrated that NUAK1 is a novel target gene for miR-203 in HNSCC cells.

### NUAK1 is involved in invasion and EMT induction in HNSCC

**Figure 6:**
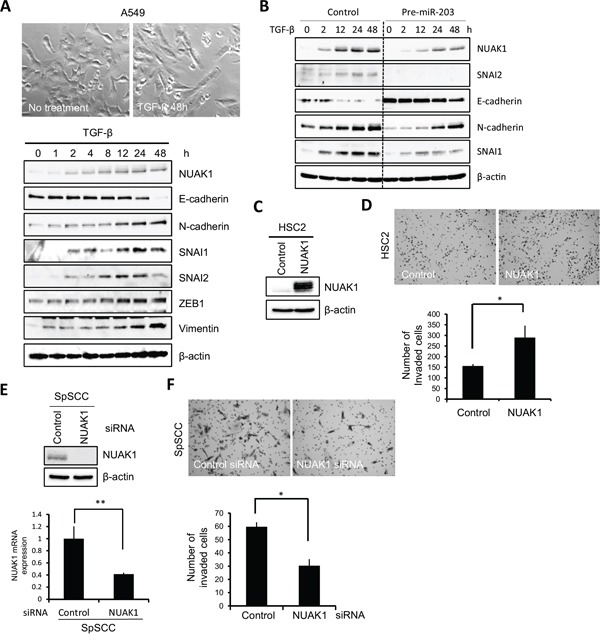
Role of NUAK1 in invasion and EMT induction **A.** A549 cells were treated with 10 ng/mL of TGF-β. The upper panel shows the cell shape at 0 and 48 h after TGF-β treatment. The lower panel shows expression of NUAK1, E-cadherin, N-cadherin, SNAI1, SNAI2, ZEB1, and vimentin. Expression of indicated proteins was examined by western blotting at 0, 1, 2, 4, 8, 12, 24, and 48 h after TGF-β treatment. β-actin was used as a control. **B.** A stable clone of pre-miR-203-transfected A549 cells was obtained. Control- or pre-miR-203-transfected A549 cells were treated with 10 ng/mL of TGF-β. Expression of the indicated proteins was examined by western blotting at 0, 2, 12, 24, and 48 h after TGF-β treatment. β-actin expression was used as a loading control. **C.** NUAK1 or empty vector was transfected into HSC2 cells. The expression of NUAK1 was examined by western blotting. β-actin was used as a loading control. **D.** The invasiveness of NUAK1-overexpressing HSC2 cells was examined by *in vitro* invasion assay for 10 h. The upper panel shows that the cells penetrated to the lower side of the membrane. The lower graph shows the results of the *in vitro* invasion assay presented as means ± SD. *P < 0.05. **E.** SpSCC cells were transfected by NUAK1 siRNA or control siRNA. A scrambled sequence that did not show significant homology to rat, mouse or human gene sequences was used as a control. The effect of knockdown was evaluated by western blotting and real-time PCR, and β-actin and GAPDH were used as loading controls, respectively. **P < 0.01. **F.** The invasiveness of NUAK1-knocked-down SpSCC cells was examined by *in vitro* invasion assay for 9 h. The upper panel shows that the cells penetrated to the lower side of the membrane. The lower graph shows the results of *in vitro* invasion assay presented as means ± SD. *P < 0.05.

To determine the involvement of NUAK1 in invasion and EMT induction, we examined NUAK1 expression in an EMT induction model using A549 cells. As far as we know, there is no EMT induction model using HNSCC cells. Therefore, we used NMuMG cells and A549 cells as an EMT-inducing model in this study. After 48 h of TGF-β treatment, the cells showed EMT features such as spindle shape, downregulation of E-cadherin, and upregulation of N-cadherin, SNAI1, SNAI2, ZEB1, and vimentin (Figure 6A). NUAK1 expression increased in a time-dependent way after TGF-β treatment (Figure 6A). We also confirmed that increased NUAK1 expression was observed in another EMT induction model using NMuMG cells (Supplemental Figure 5). Moreover, we examined NUAK1 expression in stable pre-miR-203-infected A549 cells after TGF-β treatment. In stable pre-miR-203-infected A549 cells, miR-203 delayed the EMT induction shown by downregulation of E-cadherin and upregulation of N-cadherin after TGF-β treatment (Figure 6B). Interestingly, increased expression of NUAK1 and SNAI2 was inhibited in stable pre-miR-203-infected A549 cells after TGF-β treatment (Figure 6B), suggesting that miR-203 downregulation may be important for EMT induction via NUAK1 upregulation. However, ectopic overexpression of NUAK1 itself did not induce EMT (data not shown).

To determine the role of NUAK1 in the invasion of HNSCC cells, we transfected NUAK1 into HSC2 cells with low NUAK1 expression levels. In the HSC2 cells, NUAK1 overexpression promoted invasion (Figure 6C and 6D). In contrast, we transfected NUAK1 siRNA into SpSCC cells with NUAK1 expression, and found that NUAK1 siRNA suppressed the invasion of SpSCC cells (Figure 6E and 6F). Overexpression or knockdown of NUAK1 did not change cell morphology (data not shown). These findings indicate that NUAK1 is involved in the invasion of HNSCC cells.

### Downregulation of miR-203 is caused by hypermethylation in HNSCC

**Figure 7:**
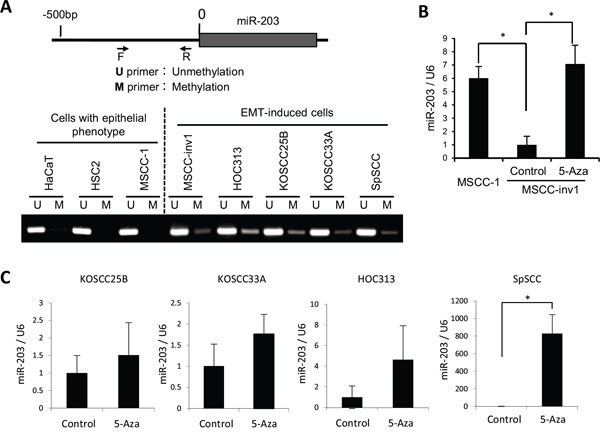
Downregulation of miR-203 by hypermethylation **A.** Analyzed regions of miR-203 are shown in the upper panel. The genomic DNA was isolated, treated with bisulfite, and then subjected to MS-PCR in HaCaT and HNSCC cells (HSC2, MSCC-1, MSCC-inv1, HOC313, KOSCC25B, KOSCC33A, and SpSCC). The primer sets that were specific for the methylated CpG (M) and the unmethylated CpG (U) were used for MS-PCR. The lower panel shows methylation status of miR-203 in cells with epithelial phenotype (HaCaT, HSC2, and MSCC-1) and EMT-induced cells (MSCC-inv1, HOC313, KOSCC25B, KOSCC33A, and SpSCC). **B.** To demethylate, MSCC-inv1 cells were treated with 5-aza-dC. Expression of miR-203/U6 was examined by real-time PCR, in comparison with MSCC-1 and MSCC-inv1 cells without 5-aza-dC treatment. The results are presented as means ± SD. *P < 0.05. **C.** To demethylate, KOSCC25B, KOSCC33A, HOC313, and SpSCC cells were treated with 5-aza-dC. Expression of miR-203/U6 was examined by real-time PCR. The results are presented as means ± SD. *P < 0.05.

Because miR-203 is downregulated by promoter hypermethylation in some human cancers [29–32], we tested whether this was also the case in HNSCC cells. The genomic DNA was isolated, treated with bisulfite, and then subjected to methylation-specific PCR (MS-PCR). Interestingly, a methylated band was observed in all HNSCC cells with an EMT-induced phenotype, but not in cells with an epithelial phenotype (Figure 7A). Moreover, demethylation by 5-aza-2′-deoxycytidine (5-aza-dC) treatment increased miR-203 expression in MSCC-inv1 cells compared with MSCC-1 cells (Figure 7B). In other HNSCC cells with an EMT-induced phenotype, 5-aza-dC treatment also increased miR-203 expression (Figure 7C). In 5-aza-dC-treated HNSCC cells, NUAK1 expression was not always downregulated (Supplemental Figure 6). SNAI2 expression was downregulated by 5-aza-dC treatment (Supplemental Figure 6). Collectively, these findings show that the expression of miR-203 was downregulated in HNSCC cells with an EMT-induced phenotype mainly by promoter hypermethylation.

### NUAK1 expression is well correlated with the invasion pattern in HNSCC

**Figure 8:**
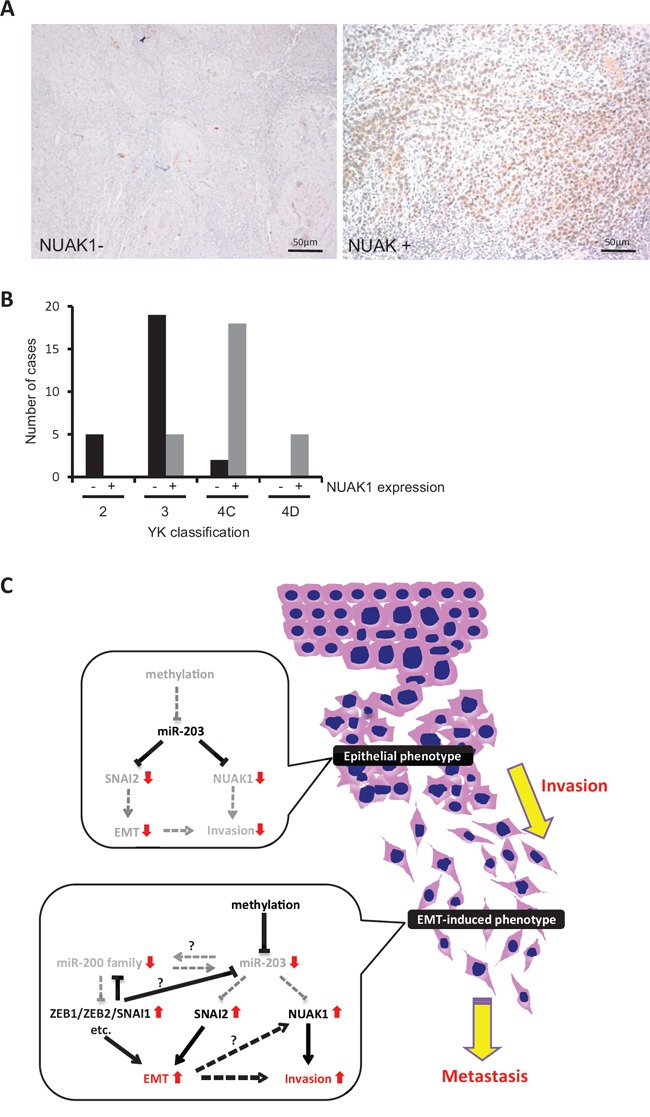
NUAK1 expression and its correlation with invasion pattern in HNSCC cases **A.** NUAK1 expression was examined by immunohistochemistry in 54 HNSCC cases. Representative pictures of NUAK1 positive or negative HNSCC case are shown. **B.** The graph shows the number of cases with or without NUAK1 expression in each grade of the YK classification (YK-2, -3, -4C, and -4D). **C.** Schematic model of the role of miR-203 in HNSCC. In HNSCC cells with epithelial phenotype, miR-203 suppresses the expression of NUAK1 and SNAI2. In HNSCC cells with EMT phenotype, miR-203 is downregulated by hypermethylation. Therefore, miR-203 cannot suppress the expression of NUAK1 and SNAI2. Elevated expression of NUAK1 may induce the invasion of HNSCC cells. In addition, the miR-200 family (miR-200a, -200b, -200c, and -141) is also downregulated and target genes including ZEB1, ZEB2, SNAI1, etc. are upregulated. Downregulation of miR-203 may be caused by downregulation of the miR-200 family and/or upregulation of SNAI1/SNAI2 and ZEB1/ZEB2. Elevated expression of NUAK1, ZEB1, ZEB2, SNAI1, and SNAI2 may induce EMT in HNSCC cells.

We examined NUAK1 expression and its correlation with miR-203 and malignant behaviors including invasion pattern and lymph node metastasis in HNSCC cases. To determine the correlation between NUAK1 and miR-203, we examined the expression of NUAK1 mRNA and miR-203 in 33 HNSCC cases. In Supplemental Figure 7A, the graph shows the expression ratio of NUAK1/GAPDH and miR-203/U6 in each HNSCC case. In 16 of the 33 (48%) cases, an inverse correlation between NUAK1 and miR-203 was observed. In 13 of the 16 (81%) cases with inverse correlation, high NUAK1 expression and low expression of miR-203 were observed. Moreover, we examined NUAK1 expression and its correlation with malignant behaviors including histological differentiation, invasion pattern, and lymph node metastasis in 54 HNSCC cases by immunohistochemistry. For evaluating the invasive pattern, we used the Yamamoto-Kohama (YK) classification. NUAK1 expression was observed in 28 of the 54 (52%) HNSCC cases (Figure 8A and Table 1). Interestingly, NUAK1 expression was well correlated with histological differentiation, invasion pattern, and lymph node metastasis (Table 1). In brief, NUAK1 expression was well correlated with poor differentiation, aggressive invasion patterns (YK-4C and -4D), and lymph node metastasis (Figure 8B, Table 1, and Supplemental Figure 7B).

**Table 1:** Summary of NUAK1 expression in HNSCC cases

		Total	NUAK1 expression		*P* value
			−	+	
Differentiation	Well Moderate Poor	24 24 6	17(71%) 9(38%) 0(0%)	7(29%) 15(63%) 6(100%)	0.00002
Pattern of invasion	2 3 4C 4D	5 24 20 5	5(100%) 19(79%) 2(10%) 0(0%)	0(0%) 5(21%) 18(90%) 5(100%)	0.00001
Lymph node metastasis	− +	24 30	18(75%) 8(27%)	6(25%) 22(73%)	0.0003
Total		54	26(48%)	28(52%)	

## DISCUSSION

In this study, we identified miR-203 and the miR-200 family (miR-200a, -200b, -200c, and -141) as downregulated miRNAs by comparing miRNA expression profiles between a parent cell line and its clone with highly invasive capability and EMT features. Members of the miR-200 family are well known to be the most altered miRNAs during EMT [9]. In addition, miR-203 downregulation has been described in various cancers including esophagus, cervix, prostate, breast, and liver cancer, and miR-203 inhibits cancerous invasion by targeting SNAI1, SNAI2, ZEB2, and VEGFA [23–28,32–35]. The miRNAs identified in this study were commonly downregulated in HNSCC cell lines with EMT features (Figure 2B). It is known that these miRNAs and their target genes including ZEB1/ZEB2 and SNAI1/SNAI2 are important factors for determining the phenotype of cells (epithelial or mesenchymal) [11,12,23–26,36]. Therefore, these observation and our findings lead us to hypothesize that the miR-200 family and miR-203 cooperatively maintain the epithelial state through a feedback loop via suppression of SNAI1/SNAI2 and ZEB1/ZEB2. Actually, ectopic expression of miR-203 and the miR-200 family caused restoration of E-cadherin (Supplemental Figure 1B), and long-term induction of pre-miR-203 caused MET in MSCC-inv1 cells (Figure 3C and 3D). Moreover, transient induction of miR-203 upregulated expression levels of the miR-200 family (miR-200b, -200c, and -141) (data not shown). These data suggest the existence of a network comprising the miR-200 family and miR-203. In transient induction of EMT, downregulation of miR-203 may be caused by downregulation of the miR-200 family and/or upregulation of SNAI1/SNAI2 and ZEB1/ZEB2 (Figure 8C). To prove this, further analyses are necessary.

Because the miR-200 family has been well studied in the field of cancer, we focused here on the role of miR-203 in EMT induction and invasion in HNSCC. Indeed, we demonstrated that miR-203 was downregulated during EMT induction by TGF-β, and that miR-203 suppressed invasion of HNSCC cells. To identify the target genes of miR-203 involved in cancerous invasion, we performed microarray analysis using the total RNA prepared from extracts of the miR-203-transfected cells immunoprecipitated with anti-Ago2 antibody. This method can cyclopedically search a target gene in RISC. In fact, SNAI2 and VEGFA, which have been identified as targets of miR-203 [25–28], were included in the extracts of the miR-203-transfected cells immunoprecipitated with anti-Ago2 antibody. Therefore, we thought that this method would be useful for searching the target genes. Here we identified NUAK1, which is involved in EMT induction and aggressive cancerous invasiveness of HNSCC cells, as a novel target gene. However, during the preparation of this manuscript, it has been reported that NUAK1 is a target gene of miR-203 for suppressing prometastatic activity via cell metabolism [37]. The researchers also identified LASP1 and SPARC as target genes of miR-203 for the involvement of cytoskeletal dynamics and extracellular matrix remodeling [37]. Previous reports have suggested that miR-203 promotes epithelial differentiation by suppressing ΔNp63 and stemness related factor Bmi1 [38,39]. It is widely accepted that cancer cells undergoing EMT become more “stem-like” [40]. In this study, during the miR-203 target search, we selected molecules related to cancerous invasion or EMT, but many transcription factors or chromatin remodeling-related genes are detected by Ago2-IP microarray (Supplemental Table 1). There may be other target genes of miR-203 that are involved in EMT and invasion in HNSCC. These findings suggest that miR-203 is a tumor suppressor miRNA that works by suppressing cell metabolism, stemness, EMT induction, and cancerous invasion. Importantly, hypermethylation of the CpG islands existing upstream on the miR-203 chromosome is frequently found in various cancers including HNSCC (Figure 7) [30,32].

NUAK1 is a member of AMP-activated protein kinase (AMPK) catalytic subunit family [41]. AMPKs are highly conserved molecules that work as metabolic sensors, and their activity has been linked to the regulation of metabolism and to the maintenance of polarity under stress conditions [41]. NUAK1 is associated with cancerous invasion in breast and lung cancers, colorectal carcinoma, and multiple myeloma [42–45]. Here we demonstrated that NUAK1 is involved in the invasion and EMT induction of HNSCC. However, NUAK1 overexpression and knockdown did not change the cell morphology or the expression of EMT-related molecules including E-cadherin, N-cadherin, vimentin, SNAI1, and SNAI2 (data not shown). This finding indicates that NUAK1 itself cannot induce EMT. As with SNAI1 and SNAI2, NUAK1 may be upregulated during EMT, and subsequently involved in the invasion of HNSCC cells (Figure 8C). Previous studies have shown that NUAK1 activates MT1-MMP, which results in promoting the metastasis of breast and pancreatic cancer via activation of MMP-2 and MMP-9 [42,46], so miR-203-driven invasion of HNSCC cells may be regulated by a pathway that is distinct from EMT induction. Moreover, NUAK1 inhibits apoptosis and promotes proliferation and Myc expression in colon, liver, and lung cancer [47]. In this study, NUAK1 siRNA did not influence the proliferation of HNSCC cells (data not shown). Importantly, NUAK1 expression was well correlated with histological differentiation, invasion pattern, and lymph node metastasis. Therefore, we believe that NUAK1 could be used as a novel marker for the prediction of malignant behavior in HNSCC. We expect that miR-203 may become a therapeutic drug for the inhibition of NUAK1 expression in the treatment for HNSCC.

## MATERIALS AND METHODS

### Plasmid construction

Human NUAK1 cDNA was isolated from the cDNA of the SpSCC cells by RT-PCR using sense and antisense primers. NUAK1 cDNA was then subcloned by insertion into the EcoRI restriction site of the pBICEP-CMV2 expression vector (Sigma-Aldrich). The NUAK1-pBICEP-CMV2 plasmid or the vector alone was introduced into target cells using an X-tremeGENE HP DNA Transfection Reagent (Roche Applied Science).

### Cell culture and proliferation assay

MSCC-1 and MSCC-inv1 cells were previously established in our laboratory [13,14]. SpSCC cells were established from spindle cell carcinoma arising in the gingiva [48]. HeLa, 293T, HSC2, A549, and NMuMG cell lines were obtained from the American Type Culture Collection and Riken Bioresource Center Cell Bank (Japan). The HOC313 cell line was provided by Dr. Kamata (Hiroshima University, Japan). The KOSCC25B and KOSCC33A cell lines were provided by Dr. Sam-Pyo Hong (Seoul National University, Korea). The HaCaT cell line was provided by Dr. P. Boukamp (German Cancer Research Center, Germany). MSCC-1 and MSCC-inv1 cells were maintained in Keratinocyte-SFM (Invitrogen). HSC2, KOSCC25B, and KOSCC33A cells were maintained in RPMI-1640 medium (Nissui Pharmaceutical Co. Ltd.) supplemented with 10% heat-inactivated FBS (Invitrogen) and 100 U/mL penicillin-streptomycin (Sigma-Aldrich) in 5% CO_2_ at 37°C. SpSCC, HeLa, 293T, A549, NMuMG, HOC313, and HaCaT cells were maintained in Dulbecco's Modified Eagle's Medium (Nissui Pharmaceutical Co. Ltd.) supplemented with 10% heat-inactivated FBS and 100 U/mL penicillin-streptomycin in 5% CO_2_ at 37°C. For EMT induction, NMuMG and A549 cells were serum starved for 24 h and treated with 10 ng/mL human recombinant TGF-β1 (Pepro Tech) for the indicated time. For the proliferation assay, 5000 cells were plated onto a 24-well plate and incubated for 0, 2, 4, and 6 days (n = 3). The cells were then trypsinized and counted using a cell counter (Coulter Z1, Beckman-Coulter). The average numbers of cells were compared and statistical assessment was carried out by the Mann-Whitney U-test. A *P-*value of < 0.05 was considered statistically significant.

### miRNA microarray analysis

To compare miRNA expression profiles between MSCC-1 and MSCC-inv1, total RNAs extracted from each cell line using a mirVANA^TM^ miRNA Isolation Kit (Ambion) were analyzed with a 3D-Gene Human miRNA oligo chip (TORAY). The RT reaction was performed using Megaplex^TM^ RT primers and human pool A, B (Applied Biosystems). The obtained cDNA was amplified with TaqMan^®^ Human MicroRNA Array A, B using Real time PCR ABI7900HT (Applied Biosystems). The amplification reaction was monitored using SDS software v2.3 and the data were analyzed with RQ Manger 1.2.

### Microarray analysis of immunoprecipitation products using anti-Ago2 antibody

Control- or miR-203-transfected cells were collected using a microRNA Isolation Kit, Human Ago2 (Wako), and miRNAs were isolated and immunoprecipitated with anti-Ago2 antibody. The purified miRNAs and target mRNAs, which form a complex with Ago2, were then obtained. The isolated mRNAs were analyzed using a 3D-Gene Human Oligo chip 25k (TORAY).

### RT-PCR

Total RNA was isolated from tumor cells using an RNeasy Mini Kit (Qiagen). These isolates were quantified and their purity was evaluated using a spectrophotometer. The cDNA was synthesized from 1 μg of total RNA according to ReverTra Dash (Toyobo Biochemicals). The primer sequences are shown in Supplemental Table 2. Total cDNA was amplified using Go Taq^®^ Green Master Mix (Promega) in a My Cycler Thermal Cycler (Bio-Rad) for 25-30 cycles of denaturation at 94°C for 30 s, annealing at 60°C for 30 s, and extension at 72°C for 30 s (for all primers). The amplicons were resolved on 1.2% agarose/TAE gels (Nacalai tesque Inc.) at 100 mV and visualized by ethidium bromide staining.

### Real-time PCR

To evaluate miRNAs, total RNA was isolated from cells using the miRNeasy Mini Kit (Qiagen). These isolates were quantified and their purity was evaluated using a spectrophotometer. The cDNA was synthesized from 10 ng of total RNA using a TaqMan^®^ MicroRNA Assay and a TaqMan^®^ MicroRNA Reverse transcription Kit (Applied Biosystems). Synthesized cDNA was amplified with a TaqMan^®^ MicroRNA Assay and a TaqMan^®^ Universal Master Mix II, no UNG (Applied Biosystems) using a Step One Plus Real-Time PCR system (Applied Biosystems) for 45 cycles of denaturation at 95°C for 15 s, and annealing and extension at 60°C for 60 s. Data were normalized with U6 and each analysis was performed three times. The average of three trials was used for statistical analysis. ANOVA test was used for comparison of variables in more than two groups and a t-test was used for two groups. A *P*-value < 0.05 was considered statistically significant.

To evaluate mRNAs, the cDNA was synthesized from 1 μg of total RNA and amplified with KOD SYBR^®^ qPCR Mix (Toyobo Biochemicals) and using a Step One Plus Real-Time PCR system (Applied Biosystems) for 40 cycles of denaturation at 98°C for 10 s, annealing at 60°C for 10 s, and extension at 68°C for 30 s. The primer sequences are shown in Supplemental Table 2. Data were normalized with GAPDH and each analysis was performed three times. The average of three trials was used for statistical analysis. The ANOVA test was used for comparison of variables in more than two groups and the t-test was used for two groups. A *P*-value < 0.05 was considered statistically significant.

### Western blotting analysis

Sample protein concentrations were measured by the Bradford protein assay (Bio-Rad), and 20 μg total protein/lane was subjected to electrophoresis on 10% polyacrylamide gels followed by electroblotting onto nitrocellulose filters. The membranes were blocked with 3% milk in TBS-T and incubated overnight at 4°C with the following antibodies: anti-NUAK1 polyclonal antibody (Cell Signaling Technology, #4458), anti-E-cadherin monoclonal antibody (BD Transduction Laboratories, 610181), anti-N-cadherin monoclonal antibody (BD Transduction Laboratories, 610920), anti-SNAI1 polyclonal antibody (Cell Signaling Technology, #3879), anti-SNAI2 polyclonal antibody (Cell Signaling Technology, #9585), anti-ZEB1 antibody (Santa Cruz Biotechnology, sc-25388), anti-vimentin monoclonal antibody (Dako, #M0725), and anti-β-actin polyclonal antibody (Sigma-Aldrich, A5441). The membranes were then washed with TBS-T and incubated with specific secondary antibodies, and the proteins were visualized using the ECL western blotting detection system (GE Healthcare).

### *In vitro* invasion assay

The membrane of a 24-well cell culture insert with 8 μm pores (BD Transduction Laboratories) was coated with 20 μg of Matrigel (BD Transduction Laboratories). 1.5 × 10^5^ cells were resuspended in 100 μL of medium and placed in the upper compartment of the cell culture insert. The lower component was filled with 500 μL of medium. After incubation at 37°C for the indicated time, the cells that penetrated the membrane into the lower side were fixed with formalin, and were stained with hematoxylin. Cells were counted in three individual fields on each insert. The average was used for the t-test. A *P*-value < 0.05 was considered statistically significant.

### Transient miRNA/siRNA transfection

A hsa-miRNAs duplex was obtained from Cosmo Bio (miCENTURY OX miNatural, hsa-miR-203). Anti-miR-203 (Ambion^®^ Anti-miR™ miRNA Inhibitor, product number AM10152) was used as a miR-203 inhibitor. Anti-miR-203 is a single-stranded nucleic acid and is designed to specifically bind to and inhibit endogenous miRNA molecules. Because anti-miR-203 downregulates miRNA activity, the mRNA level of miR-203 does not always reflect the effect of the inhibitor. In this study, we confirmed upregulation of SNAI2 and VEGFA mRNA as a known target of miR-203 (Figure 4D). Cells were transfected with 5 nM hsa-miRNAs and with 5-100 nM anti-miR-203 using Lipofectamine RNAiMax (Invitrogen). siRNAs targeting NUAK1 and negative control siRNA were purchased from Santa Cruz Biotechnology. The siNUAK1 used in this study contained three to five target-specific 19-25 nt siRNAs designed to knockdown gene expression. Cells were transfected with 35 nM siNUAK1 using X-tremeGENE HP (Roche Applied Science). After incubation for 48 h, the cells were used for each analysis.

### Lentivirus infection

Lentiviruses were generated by cotransfecting 0.9 μg of lentiviral vector (premiR-203 or scramble negative control vectors, System Biosciences) and 2.7 μg of packaging plasmid mix (1:1:1 for 0.9 μg pPACK-H1-GAG, pPACK-H1-Rev, and pVSV-G) in 293T cells using Lipofectamine LTX Plus reagent (Invitrogen). Supernatants were collected 48 h after transfection, filtered through a 0.45-μm membrane, and directly used to infect cells. Two weeks after infection of lentiviruses containing pre-miR-203 or scramble negative control vectors with GFP, GFP-expressing cells were sorted by flow cytometry to obtain stable pre-miR-203-expressing cells.

### Luciferase reporter assay

The sense and antisense oligonucleotides for the putative miR-203 binding site at the 3′-UTR of SNAI2 were annealed and cloned into a pmirGLO vector (Promega). The oligonucleotides had the following sequences:

SNAI2-1: 5′-CATTGCTGCCAAATCATTTCAA-3′
SNAI2-2: 5′-TTACTATTTTAAAACATTTTAA-3′
SNAI2-3: 5′-TAATGTACTTAAACTATTTCAA-3′

The full-length 3′-UTR of human NUAK1 was amplified by PCR (Supplemental Table 2) from genomic DNA and cloned at the EcoRI site into the pmirGLO vector (Promega). These constructs were confirmed by sequencing. For luciferase activity analysis, these vectors were cotransfected with the miRNA duplex in 293T using DharmaFECT Duo Transfection Reagent (Thermo Fisher Scientific) for 72 h, and luciferase assays were performed with the Dual-Luciferase reporter system (Promega). Luminescent signals were quantified by luminometer (Glomax; Promega), and each value from the firefly luciferase construct was normalized by *Renilla* luciferase activity. The average of three individual trials was used for the t-test. A *P*-value < 0.05 was considered statistically significant.

### Tissue samples

For immunohistochemical examination, 54 tissue samples of HNSCC were retrieved from the Surgical Pathology Registry of Hiroshima University Hospital from 1998 to 2004, after approval by the Ethical Committee of our institutions. Cases of T2 were used for analysis and 10% buffered formalin-fixed and paraffin-embedded tissues were used. The histological grade and stage of the tumors were classified according to the criteria of the Japan Society for Head and Neck Cancer. For the invasion pattern, we used the Yamamoto-Kohama (YK) classification, which is based on histological architecture and mode of invasion. The YK classification is categorized into 5 grades: grade 1, well defined borderline; 2, cords, less marked borderline; 3, groups of cells, no distinct borderline; 4C, diffuse invasion with cord-like type; and 4D, with diffuse type invasion [49].

For real-time PCR analysis, 33 patients (16 men, 17 women; 44-91 years of age, mean: 67.1 years) with primary HNSCCs, who received treatment at Nara Medical University Hospital, Kashihara, Japan between 2003 and 2006, were randomly selected. All cases were performed without pre-operative therapy before surgery and sample preparation took place after approval by the Medical Ethical Committee of the Nara Medical University.

### Immunohistochemistry

Tissue sections were deparaffinized in xylene and rehydrated in descending grades of ethanol. Endogenous peroxidase activity was blocked with methanol containing 0.3% H_2_O_2_ for 30 min. Antigen retrieval was carried out by Pascal Pressure Chamber (Dako) using Dako Target Retrieval Solution, pH 9.0 (Dako) for 30 s at 125°C. After treatment with casein for prevention of non-specific background for 10 min, the sections were treated with polyclonal anti-NUAK1 antibody (Cell Signaling Technology, 1:50) using a MI-77 microwave oven (Azumaya Company) for 30 min (4 s on and 4 s off) followed by incubation at 4°C overnight. After incubation with secondary antibody, the reaction was detected by 3,3′-diaminobenzidine (Dako). The sections were then counterstained with hematoxylin, and dehydrated in ascending grades of ethanol, and finally the slides were mounted. Correlation between the immunohistochemical staining of NUAK1 and histological differentiation, pattern of invasion, and lymph node metastasis was analyzed and the significance was validated by the Fisher's exact test.

### Analysis of the methylation status

DNA was extracted and purified from cells using a DNeasy^®^ Tissue Kit (Qiagen) and 500 ng DNA was bisulfited with a MethylCode^TM^ Bisulfite Conversion Kit (Invitrogen) and amplified by HotStarTaq^®^ DNA polymerase (Qiagen) and U and M primers; the U primer detected unmethylated sequences and the M primer detected methylated sequences (Supplemental Table 2). Amplification was performed for 40 cycles of denaturation at 94°C for 30 s, annealing at 54°C for 30 s, and extension at 72°C for 60 s. The amplicons were resolved on 1.5% agarose/TAE gels (Nacalai Tesque) at 100 mV and visualized by ethidium bromide staining. To demethylate the target cells, cells were incubated with the medium containing 5 μM 5-asa-2′-deoxyxytidine (5-aza-dC) (Sigma-Aldrich) for 48 h or the indicated time.

## SUPPLEMENTARY FIGURES AND TABLES, DATAS






