# Supplementary material for: microRNA-203 suppresses invasion and epithelial-mesenchymal transition induction via targeting NUAK1 in head and neck cancer
Source: Oncotarget. 2016 Jan 22;7(7):8223–39. doi: 10.18632/oncotarget.6972 (PMC4884988; doi:10.18632/oncotarget.6972)
Supplement: Supplementary file 1 [file oncotarget-07-8223-s001.pdf]

SUPPLEMENTARY FIGURES AND TABLES

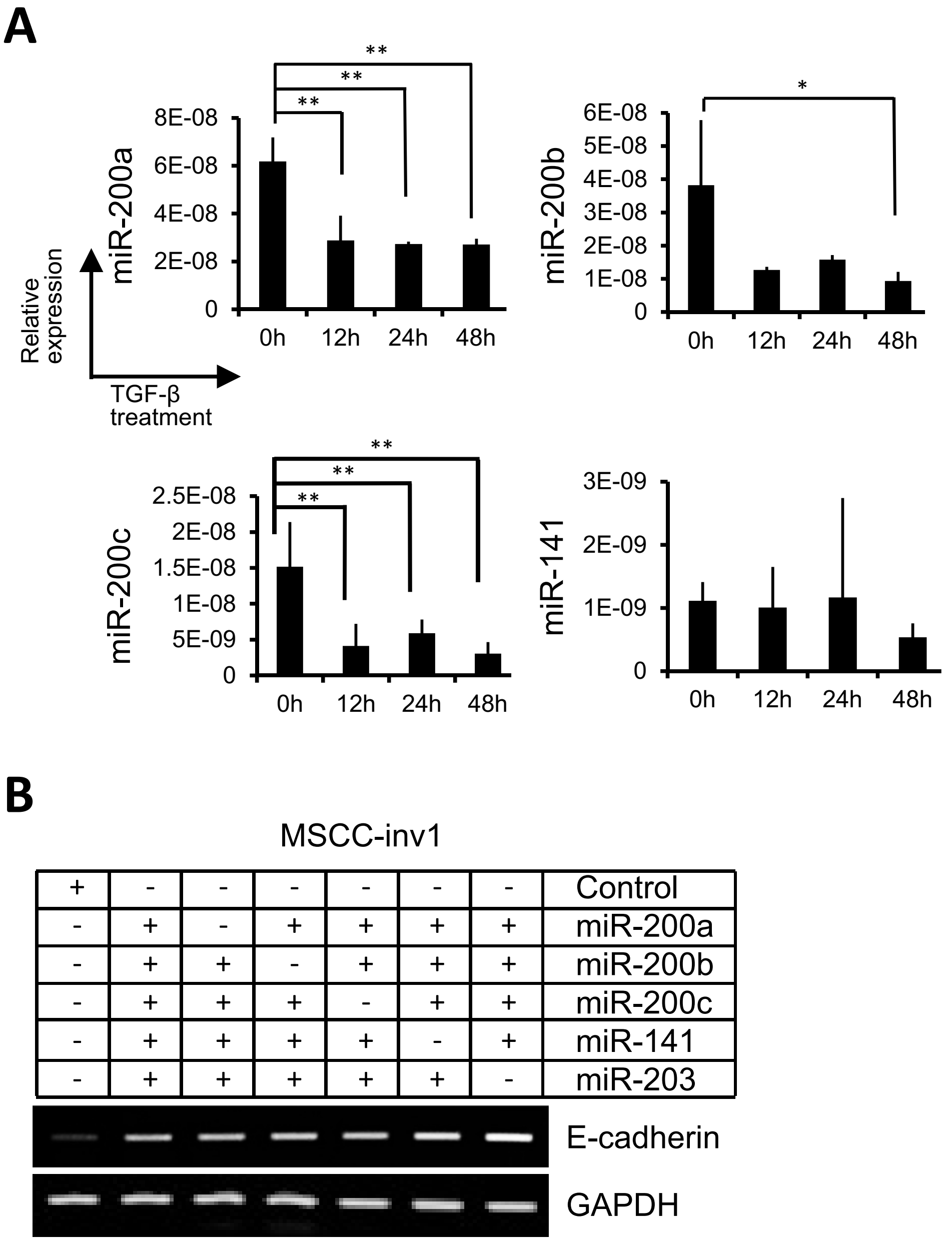

Supplementary Figure S1: A. Expression of miR-200a, -200b, -200c, and -141 was examined by real-time PCR at 0, 12, 24, and 48 h (n = 3) after 10 ng/mL of TGF-β treatment in NMuMG cells. The graph shows the expression of these miRNAs (miRNA/U6). All results are presented as means ± SD. $**P < 0.01$, $*P < 0.05$. **B.** The mature miR-200a, -200b, -200c, -141, and -203 were transfected into MSCC-inv1 cells with EMT features. Moreover, we removed one of the miRNAs from transfection with all five miRNAs (miR-200a, -200b, -200c, -141, and -203). After 72 h of transfection, the expression of E-cadherin mRNA was examined by RT-PCR. GAPDH expression was used as a control.

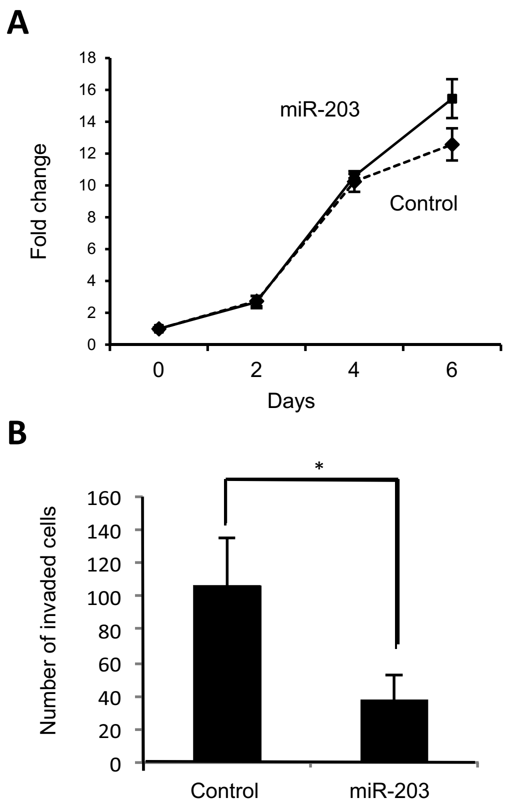

Supplementary Figure S2: A. The graph shows cell growth of control miRNA or miR-203 transfected MSCC-inv1 cells at 0, 2, 4, and 6 day (n = 3). The result is presented as means ± SD and $P < 0.05$ was considered significant. **B.** The graph shows invasion capability of control miRNA or miR-203 transfected MSCC-inv1 cells. The invasiveness of the cells was determined by *in vitro* invasion assay for 20 h. *$P < 0.05$.

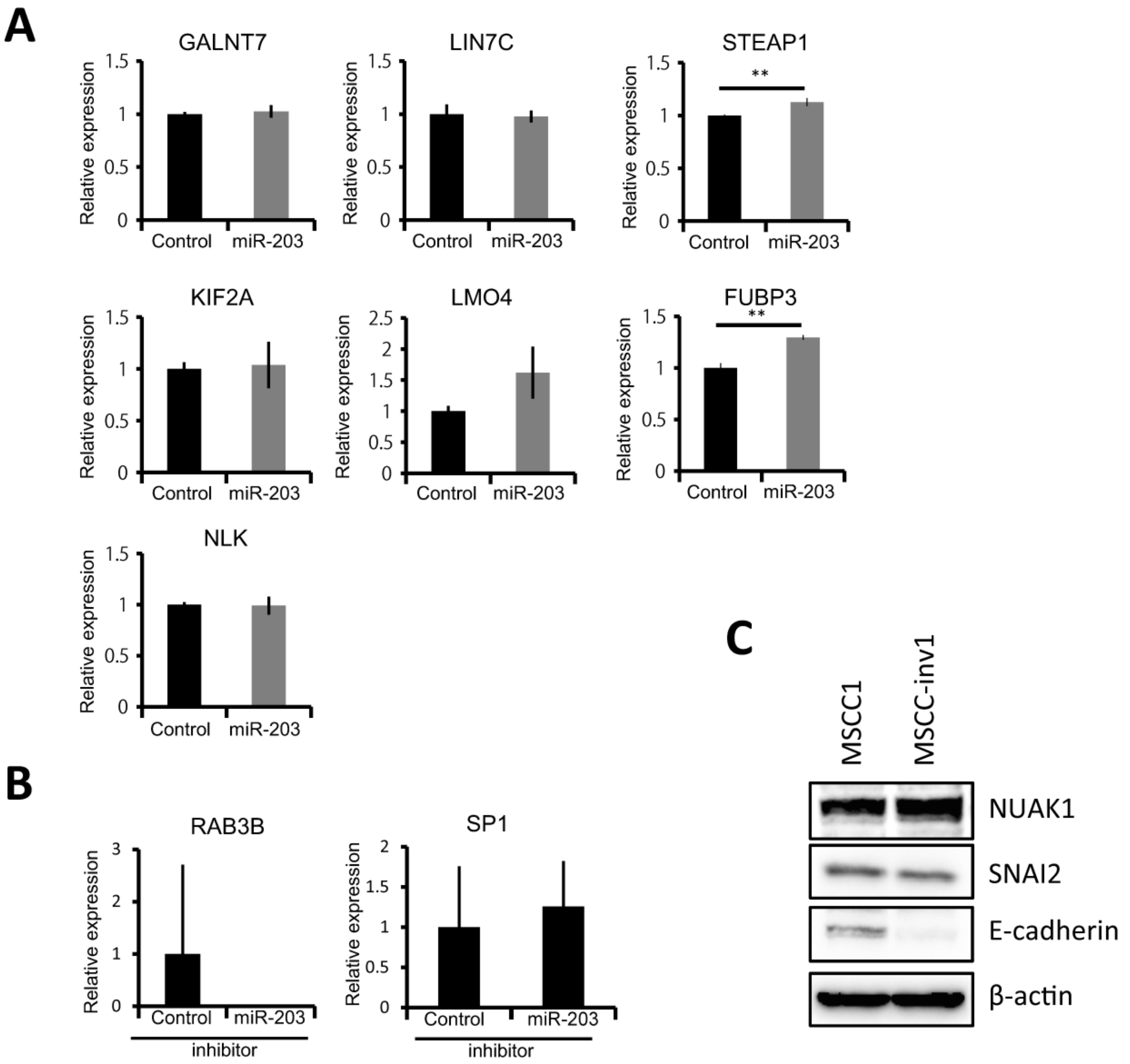

Supplementary Figure S3: A. The graph shows the expression of candidate genes of miR-203 (GALNT7, KIF2A, NLK, LIN7C, LMO4, STEAP1, and FUBP3) in control- or mature miR-203-transfected MSCC-inv1 cells. The graph shows the expression of these mRNAs (mRNA/GAPDH). The results are presented as means ± SD. ***P* < 0.01. **B.** The graph shows the expression of candidate genes of miR-203 (RAB3B and SP1) in control- or miR-203 inhibitor- transfected HSC2 cells. OCLN expression was not observed in either the control- or miR-203 inhibitor-transfected HSC2 cells. The graph shows the expression of these mRNAs (mRNA/GAPDH). The results are presented as means ± SD. **C.** Expression of NUAK1, SNAI2, and E-cadherin was examined in MSCC-1 and MSCC-inv1 cells by western blotting analysis. ß-actin expression was used as a loading control.

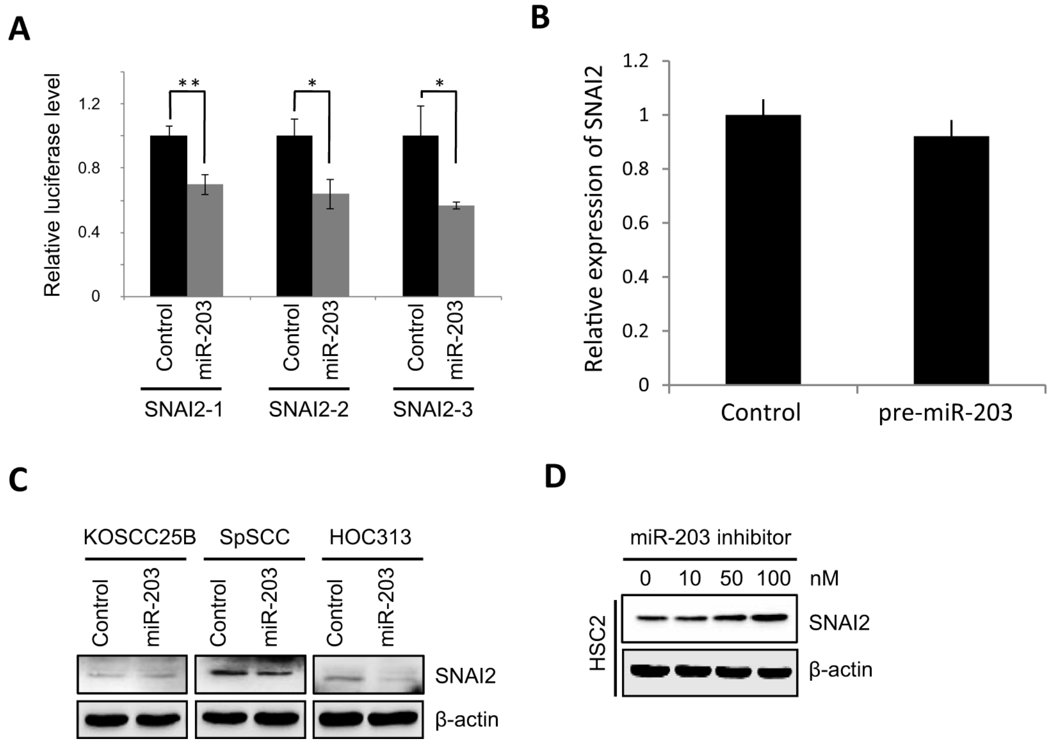

Supplementary Figure S4: A. Luciferase assays were performed with a pmirGLO vector containing the putative miR-203 binding sites of SNAI2, and the graph shows the relative luciferase activity in mature miR-203- or control miRNA-transfected cells. The data were normalized to control-transfected samples and the results are presented as means ± SD. $^{**}P < 0.01$, $^{*}P < 0.05$. **B.** Expression vector of pre-miR-203 or scramble negative control vector was transfected into MSCC-inv1 cells. Expression of SNAI2 mRNA was examined by real-time PCR in control- and pre-miR-203-infected MSCC-inv1 cells. The graph shows the expression of these mRNAs (mRNA/GAPDH). The results are presented as means ± SD. **C.** SNAI2 expression was examined in control- or mature miR-203-transfected HNSCC cells (KOSCC25B, SpSCC, and HOC313) by western blotting analysis. ß-actin was used as a loading control. **D.** SNAI2 expression was examined in control- or miR-203 inhibitor-transfected HSC2 cells by western blotting analysis. ß-actin was used as a loading control.

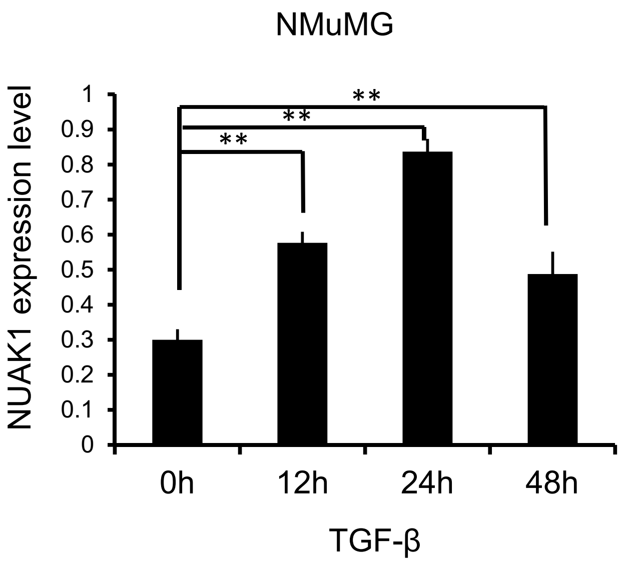

Supplementary Figure S5: Expression of NUAK1 was examined by real-time PCR at 0, 12, 24, and 48 h (n = 3) after 10 ng/mL of TGF-ß treatment in NMuMG cells. The graph shows NUAK1/GAPDH and the results are presented as means ± SD. **P < 0.01.

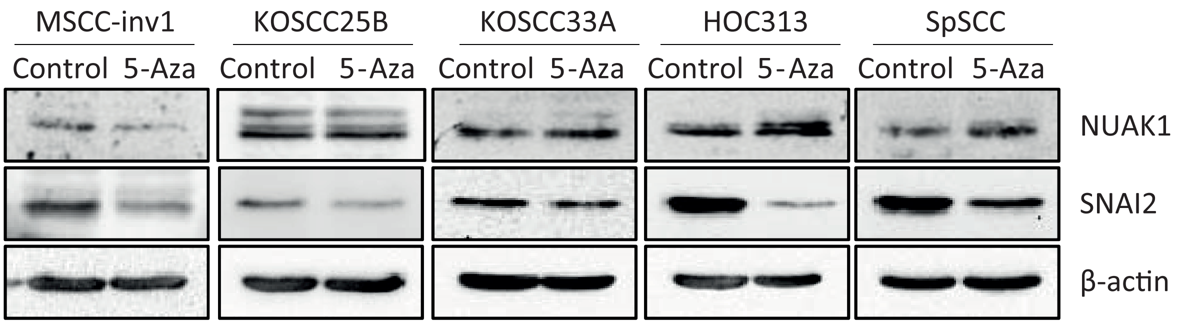

Supplementary Figure S6: Expression of NUAK1 and SNAI2 in 5-aza-dC-treated HNSCC cells. EMT-induced cells (MSCC-inv1, HOC313, KOSCC25B, KOSCC33A, and SpSCC) were treated with 5-aza-dC. NUAK1 and SNAI2 expression was examined in EMT-induced cells by western blotting analysis. ß-actin was used as a loading control for 4 d.

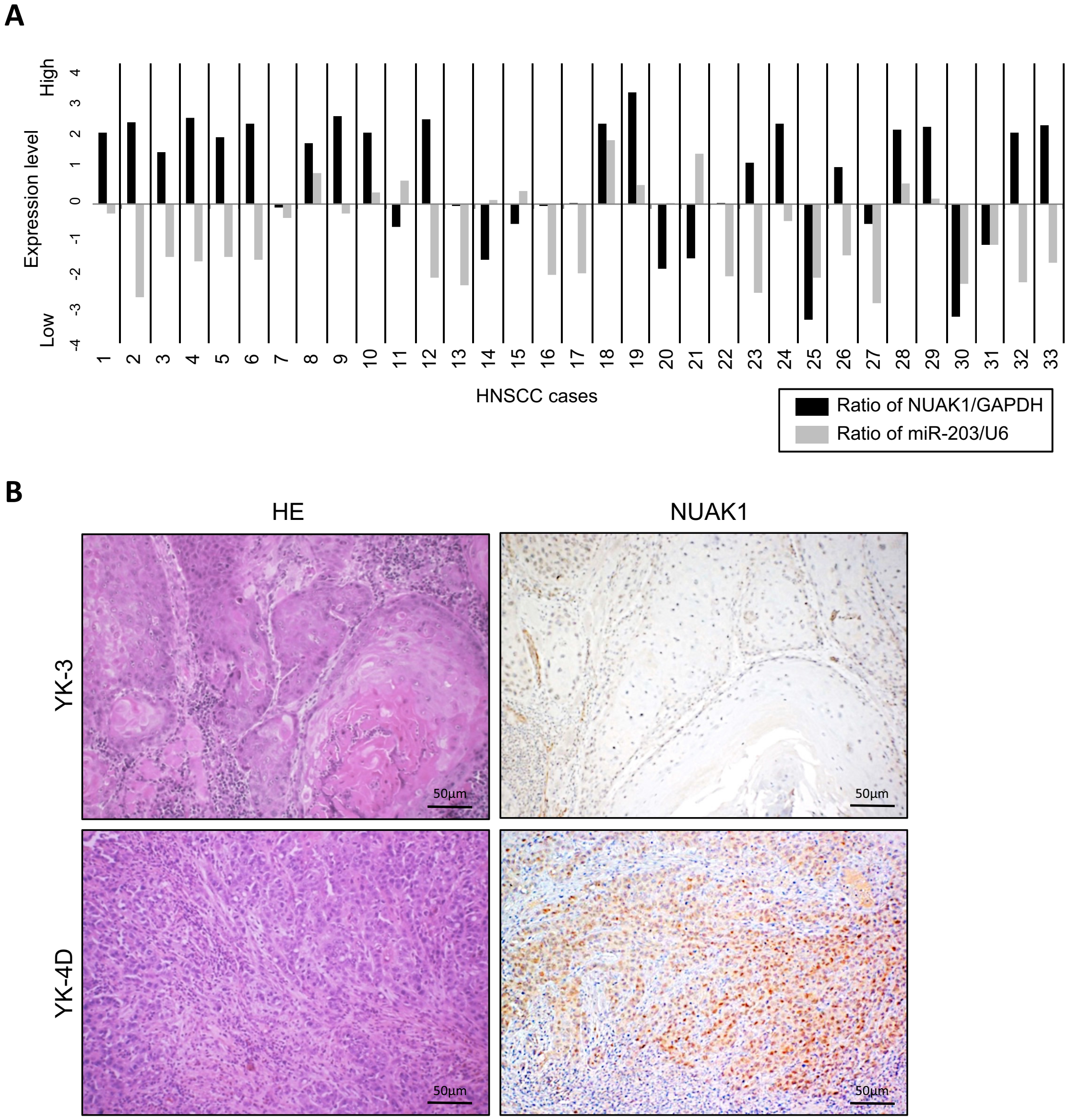

Supplementary Figure S7: A. Expression of NUAK1 and miR-203 was examined in 33 HNSCC tissues by real-time PCR. The graph shows expression ratio of NUAK1/GAPDH and miR-203/U6 in each HNSCC case. **B.** NUAK1 expression was compared with invasion pattern in 54 HNSCC cases. For evaluation of invasion pattern, we used the YK classification. Of the 54 cases, no case was classified as YK-1. Representative pictures of hematoxylin and eosin staining and NUAK1 expression in HNSCC cases with YK-3 or YK-4D classifications are shown.

Supplemental Table 1: List of candidate genes of miR-203 target

Gene symbol	Description	Previous reports: Cancer	Previous reports: Invasion	Previous reports: EMT	Ratio miR-203/ Control
ACSL3	fatty acid metabolism	●			2.8
AFF4	transcription elongation factor	●			4.7
ARID1B	chromatin-remodeling gene	●			2.9
ARID2	chromatin-remodeling	●			2.2
BPTF	chromatin-mediated regulation of transcription	●			2.2
C14orf129	negative regulator of GSK3-beta				14.6
CALML4	chondrogenesis				2.3
CASK	calcium/calmodulin-dependent serine protein kinase	●			5.3
CBL	E3 ubiquitin ligase	●			2.4
CEP68	centrosome cohesion				2.6
CHD9	regulator of the chromatin remodelling process	●			2.1
CHORDC1	ADP-dependent HSP90 interacting protein/ mouse brain development				8.0
CLSTN3	calcium-mediated postsynaptic signals	●			6.8
COL4A4	one of the six subunits of type IV collagen				2.4
COX15	the terminal component of the mitochondrial respiratory chain				4.1
CXorf23	-				12.9
DCP2	degradation of mRNAs				4.0
ELOVL6	fatty acid elongation				3.5
FAT3	tumor suppression and planar cell polarity (PCP)				2.6
FBXO44	F-box protein family	●			2.5
**FUBP3**	regulate the expression of the c-myc oncogene	●			37.2
**GALNT7**	GalNAc-transferase family	●	●		2.7
GJB6	connexin30 protein of gap junction	●			2.7
ITPR2	release of intracellular carcium	●			2.3
JOSD1	deubiquitination				2.5
KCTD9	potassium channel gene				3.2
KIAA1009	microtubule-associated ATPase involved in cell division				20.1
KIAA1147	neuritogenesis				8.8
**KIF2A**	microtubule dinamics	●	●		2.4
KLHL15	protein ubiquitination and cytoskeletal organization				7.5
LCORL	spermatogenesis, height				22.2
**LIN7C**	cellular polarity	●	●		2.1

(*Continued*)

**LMO4**	transcriptional coregulatory protein	●	●		3.6
MAGI1	scaffolding protein at cell-cell junctions				2.8
MIDN	neurogenesis, contains a Ubiquitin-like domain				2.3
**NLK**	serine/threonine protein kinase involved in Wnt/β-catenin signalling	●	●	●	2.5
**NUAK1**	tolerance to glucose starvation, suppress apoptosis	●	●		6.0
**OCLN**	integral membrane protein of TJ	●	●		2.9
PACS2	recruit Bim and Bax to lysosomes to release cathepsin B and induce TRAIL-induced apoptosis				2.5
PAPSS2	sulfate source, increase phosphorylated Smad 2/3				2.2
PITPNB	Catalyzes the transfer of PtdIns and phosphatidylcholine between membranes				2.3
**PKD2**	calcium permeable cation channel	●	●		2.0
PPM1D	PP2C family(negative regulators of cell stress response pathways)	●			5.3
PRELP	anchor basement membranes to the underlying connective tissue	●			2.2
PRKAB1	fatty acid synthesis(regulatory subunit of AMPK)	●			3.5
**RAB3B**	member RAS oncogene family	●	●		3.1
RASAL2	GAPs function as activators of Ras superfamily of small GTPases				2.2
RBM25	regulator of alternative pre-mRNA splicing, apoptosis				2.9
SHMT1	Interconversion of serine and glycine, folate metabolism	●			2.2
SLC25A36	?	●			2.0
**SNAI2**	transcriptional repressor that binds to E-box motifs	●	●	●	2.3
SNN	toxic effects of organotins				11.7
**SP1**	Transcription factor, Binds with high affinity to GC-rich motifs, cell growth, apoptosis, differentiation	●	●	●	11.3
SPTBN1	calcium-dependent movement	●			6.9
SS18L1	transcriptional co-activator	●			2.0
**STEAP1**	cell surface antigen significantly expressed at cell-cell junction	●	●		2.7
TAF9B	involved in initiation of transcription by RNA polymerase II				7.9
TUSC2	tumor suppressor				2.1
UBE2I	ubiquitin-conjugating enzyme, sumoylation	●			2.0
**VEGFA**	angiogenesis, migration, inhibit apoptosis	●	●	●	13.6
ZBTB7B	Transcriptional repressor of ECM				5.7
ZRANB2	BMP suppressor that forms a complex with Smads	●			2.7
ZXDB	promote transcription of MHC class I and II				2.6

Supplemental Table 2: List of primer sequences

Primer	F/R	Sequence	Product size (bp)
GALNT7	F<br>R	TACCTTGATGCCCACTGTGA<br>AGCATACTCCAATCCCATGC	188
LIN7C	F<br>R	CCGCACAAGGAAAGGTTAAA<br>TGGCATTGCAGCCATTAGTA	190
OCLN	F<br>R	ATGCCTAGCTACCCCCATCT<br>GAATGCCAATCCTGCATTCT	181
KIF2A	F<br>R	TGCTCACCTTTTCCTGCTTT<br>CCGTTCAATGATATGGCACA	184
LMO4	F<br>R	GGAGGCTGAGGTTGTACTGC<br>CTACATGGCTCCACGGCTAT	193
PKD2	F<br>R	AGCCTGGATGACTCTGAGGA<br>TGGCTCGCTCCATAATCTCT	199
NLK	F<br>R	CCAGGGAATCTCCTTGTGAA<br>TCCCACAGACCAGATGTCAA	186
SP1	F<br>R	ACTACTGACGCAGGCACCTT<br>CCTAGTGAAAGCCCCCTACC	188
RAB3B	F<br>R	ATGGAACTGGCAGTGATTCC<br>CACAGCTGGATGGTCCTTTT	191
NUAK1	F<br>R	CAGCGTGTGTGACTGTGATG<br>TGCATCCTGACCAGACTGAG	217
STEAP1	F<br>R	GCCCTTCAGAACTTCAGCAC<br>ATGGAAACCATTGGCAAGAC	199
SNAI2	F<br>R	GAGCATTTGCAGACAGGTCA<br>GCTTCGGAGTGAAGAAATGC	200
VEGFA	F<br>R	AAGGAGGAGGGCAGAATCAT<br>ATCTGCATGGTGATGTTGGA	226
FUBP3	F<br>R	TCAAAAGCATCAACCAGCAG<br>GAGCTCCTTGGAGGAAAGGT	244
E-cadherin	F<br>R	TGCCCAGAAAATGAAAAAGG<br>GGATGACACAGCGTGAGA	225
ZEB1	F<br>R	TGCACTGAGTGTGGAAAAGC<br>TGGTGATGCTGAAAGAGACG	225
ZEB2	F<br>R	TGCCAAGAGAGGAAGAGGAA<br>GTGTCACTGCGCTGAAGGTA	225
GAPDH	F<br>R	GCATCCTGGGCTACACTGAG<br>TCCACCACCCTGTTGCTGTA	163
hsa-miR-203 U	F<br>R	GGGTTGTGGAGGATTAGTT<br>AAACAACTAAACTCCAAACA	
hsa-miR-203 M	F<br>R	GGGTCGTGGAGGATTAGTC<br>AAACGACTAAACTCCGAACG	
NUAK1-3UTR-pcDNA	F<br>R	GCTGGATATCTGCAGCTGTGA<br>CAACAGACTGAAAAAGGAT<br>AGTCCAGTGTGGTGGCAAAAA<br>CCAAAATGAAACAAAAATC	

Supplemental data 1

See Supplementary File 1

Supplemental data 2

See Supplementary File 2
